# The Effect of Functional and Intra-Coronary Imaging Techniques on Fluoroscopy Time, Radiation Dose and Contrast Volume during Coronary Angiography

**DOI:** 10.1038/s41598-020-63791-1

**Published:** 2020-04-24

**Authors:** Fernando De la Garza-Salazar, Diana Lorena Lankenau-Vela, Bertha Cadena-Nuñez, Arnulfo González-Cantú, Maria Elena Romero-Ibarguengoitia

**Affiliations:** 1 Universidad de Monterrey, Facultad de Medicina, Especialidades Médicas. Av. Ignacio Morones Prieto 4500, Jesús M. Garza, 66238 San Pedro Garza García Nuevo Leon, Mexico; 2 Hospital Christus Muguerza Alta Especialidad: Miguel Hidalgo, 2525, Obispado, 64060 Monterrey, Nuevo Leon, Mexico; 3 Hospital Ángeles Tampico: Av. Miguel Hidalgo 5503, Choferes, 89330 Tampico, Tamaulipas Mexico

**Keywords:** Interventional cardiology, Risk factors

## Abstract

The aim was to analyze the effect of fractional flow reserve (FFR), intravascular ultrasound (IVUS) and optical coherence tomography (OCT) on fluoroscopy time (FT), radiation dose (RD) and contrast volume (CV) in patients undergoing coronary angiography. This case-control study included consecutive patients above the age of 18, who underwent coronary angiography. FT, RD, and CV after each procedure were retrospectively recorded. Multivariate models were used to demonstrate the effect of these complementary studies and other factors, on radiation and contrast exposure. A total of 1047 patients were included, 74.5% were men and the mean (SD) age was 62.4 (12.1) years. Complementary studies performed were: IVUS (n = 237), FFR (n = 56) and OCT (n = 37). FFR and IVUS had a small effect on FT (η = 0.008 *B* = 2.2, p < 0.001; η = 0.009, *B* = 2.5, p < 0.001), while OCT had no effect (η = 0.002 *B* = 2.9, p < 0.183). IVUS, FFR and OCT had no effect on the RD. IVUS did not affect contrast volume (η = 0.002 *B* = 9.4, p < 0.163) while OCT and FFR had a small effect on CV (η = 0.006 *B* = 39, p < 0.01; η = 0.008 *B* = 37, p < 0.003). The number of placed stents had a significant effect on FT *(*η = 0.192, *Β* = 4.2, *p* < *0.001)*, RD *(*η = 0.129, *Β* = 511.8, *p* < *0.001)* and CV *(*η = 0.177, *Β* = 40.5, *p* < *0.001)*. The use of complementary studies in hemodynamics did not modify the received RD and had a minor effect on FT and the CV used.

## Introduction

Coronary angiography is the gold standard for the diagnosis of coronary artery disease^1^. Over the past decade, functional and intra-coronary imaging techniques have emerged to overcome the limitations of coronary angiography. These new techniques are Fractional Flow Reserve (FFR), Intravascular Ultrasonography (IVUS) and Optical Coherence Tomography (OCT). FFR measures pressure differences across coronary artery stenosis, using a standard guide catheter with a pressure tip. It is defined as the pressure distal to stenosis divided to the pressure before the stenosis. IVUS uses an ultrasound probe and the principle of pulse-echo ultrasonography to create a plaque image giving valuable information such as plaque composition, positive remodeling, etc. and OCT creates an image of the plaque from a probe that ejects pulsating near-infrared photons^2^. FFR is of clinical importance because of its association with lower cardiovascular mortality^3^. IVUS and OCT, can aid in decision-making, guide interventions and optimize the results of percutaneous coronary intervention^4^.

Radiation and exposure to contrast medium have been associated with metabolomic changes in cardiomyocytes, endotheliopathy, atherosclerosis and contrast nephropathy^5–7^. Many individual factors associated to radiation and contrast exposure have been reported such as vascular access, age, and female sex, but the impact of the use of complementary studies such as FFR, IVUS and/or OCT has been scarcely studied^8,9^.

The aim of this study was to analyze the effect of angiographic complementary studies such as FFR, IVUS and OCT on fluoroscopy time (FT), radiation dose (RD)* and contrast volume (CV) in patients undergoing coronary angiography. Other factors such as gender, body mass index (BMI), comorbidities, coronary lesion severity, the number of placed stents, and the number of complementary studies performed were also addressed.

Footnote: *RD is a measure of air kerma (equivalent to dose to air) at the measurement reference point, defined as a position 15 cm from the isocenter (x-ray tube side) along the central axis of the C-arm.

## Materials and Methods

This study followed STROBE methodology^10^. This was an observational, retrospective case-control study that included consecutive patients undergoing coronary angiography from 2012 to 2016. The study was conducted in the Cardiology and Internal Medicine Departments of *Hospital Christus Muguerza* in Monterrey, Mexico. We included men and women above the age of 18 years, who underwent simple coronary angiography or in conjunction with one of the following: FFR, OCT, IVUS or a combination of these. Patient characteristics were obtained from medical charts and included family history of coronary artery disease, gender, age, BMI, personal history of dyslipidemia, type 2 diabetes mellitus, chronic kidney disease and arterial hypertension. Also we obtained admission diagnosis (stable and unstable angina, non-ST and ST elevation myocardial infarction, heart failure, positive ischemia test), used complementary studies (FFR, OCT, IVUS), number of placed stents, severity of coronary lesion, vascular approach, vascular approach-related complications, and in-hospital stay (days). RD in milli-gray (mGy) and FT (min) were obtained with a General Electric Innova 3100 fluoroscope; CV (mL) was extracted from medical records. We excluded patients who underwent aortocoronary bypass and those with incomplete anthropometric or procedure information. We eliminated patients with left femoral and radial access because of the small sample size.

### Statistical analysis

Continuous variables were expressed as means and standard deviations (SD) while categorical variables were expressed as frequencies and percentages. Normality was explored for continuous variables by computing skewness and kurtosis and applying the Shapiro-Wilk test. Log-normalization was used when necessary. We used two sample *t*-test and chi square for group comparisons. Linear multiple regression models were constructed to predict the effect of multiple variables on FT, RD, and CV in patients undergoing coronary angiography. We use eta-squared (*η)* as an estimation of variance of the response variable (i.e. RD), explained by the explicative covariable (i.e. FFR). The eta-squared value was computed to calculate the effect size of variables in the models; a value of <0.02 was considered small, 0.02–0.09 medium and >0.09 large. To generalize the models, we used a 10-fold cross-validation. The models were two-sided and a p value < 0.05 was considered significant. There were no missing values. Sample size for a two-sided linear multiple regression model of 15 predictors, effect size f^2^ of 0.1, α 0.01, β 0.95 was 182. We used G*Power to calculate sample size and the statistics program R.Studio v 3.4.0. and SPSS version 24.

### Ethical approval

All procedures performed in studies involving human participants were in accordance with the ethical standards of the institutional and/or national research committee and with the 1964 Helsinki declaration and its later amendments or comparable ethical standards.

### Informed consent

Ethics Committee/Institutional Review Board waived the need for informed consent as part of the study approval. This Study has obtained IRB approval from Hospital Christus Muguerza Alta Especialidad and the registration number is CMHAE-047–17.

## Results

### Population characteristics

Our hospital team performed 1550 diagnostic angiographies in the study period; we excluded 355 patients due to the lack of clinical data, 102 patients who underwent aortocoronary bypass and twenty patients with left radial or left femoral arterial access. We included 1073 patients (power > 99%) of which 799 were men (74.5%) and the mean (SD) age was 62.4 (12.1) years. Eighty one percent (81%) of the population was overweight with a mean (SD) BMI of 28 kg/m^2^ (4.2); 75.4% of patients (n = 809) had at least one comorbidity. Table 1 shows the demographic characteristics and comorbidities of the population.

**Table 1:** Demographical characteristics and risk factors of the population.

	Men (n = 799)	Women (n = 274)	p-value [95%CI]
Mean (SD) Age	60.6 (28.2)	67.5 (27.5)	0.001 [5.32,8.38]
Family History of ASCVD	102 (12.8%)	26 (9.5%)	0.161
BMI	28.2 (3.89)	27.5 (4.97)	0.028 [−1.38, −0.07]
HT	442 (55.3%)	171 (62.4%)	0.048
DLP	340 (42.6%)	126 (46%)	0.359
T2DM	276 (34.5%)	128 (46.7%)	0.001
CKD	37 (4.6%)	12 (4.4%)	1.0

Demographic characteristics and population risk factor are compared by gender. Abbreviations: ASCVD: Atherosclerotic Cardiovascular Disease, 95%CI: Mean difference 95% Confidence Interval, BMI: Body mass index, HT: arterial hypertension, DLP: dyslipidemia, T2DM: type 2 diabetes mellitus, CKD: Chronic kidney disease.

### Angiographic characteristics

The indications for angiography were: unstable angina/non-ST elevation myocardial infarction (n = 620, 57.8%), ST-elevation myocardial infarction (n = 179, 16.7%), positive ischemia test (n = 142,13.2%), stable angina (n = 56, 5.2%), heart failure (n = 40, 3.7%) or miscellaneous (n = 36, 3.4%). Table 2 shows the main detected angiographic changes. The affected arteries were: anterior descending artery (n = 541, 50.4%), circumflex artery (n = 228, 21.2%), right coronary artery (n = 279, 26%) and left main trunk (n = 18, 1.7%). Stents were implanted in 679 patients (63%), with a (SD) of 1.14 (1.2).

**Table 2:** Angiographic characteristics.

		Men (n = 799)	Women (n = 274)	p-value [95%CI]
Mean (SD) Radiation dose (mGy)		1693.8 (1670.1)	1130.2 (1171.4)	0.001 [−777.6, −349.5]
Mean (SD) Radiation time (min)		14.4 (12.2)	12 (10.8)	0.002 [−3.9, −0.88]
Mean (SD) Contrast volume (ml)		209.2 (112.9)	171.6 (100.6)	0.001 [−51.8, −23.2]
Mean (SD) Stents placed		1.21 (1.19)	0.95 (1.19)	0.003 [−0.25, −0.084]
Complementary studies	IVUS	172 (21.5%)	65 (23.7%)	>0.05
	FFR	44 (5.5%)	12 (4.4%)	
	OCT	29 (3.6%)	8 (2.9%)	
Coronary lesion severity	No lesion	111 (13.9%)	72 (26.3%)	<0.001
	Mild	40 (5%)	18 (6.6%)	
	Moderate	52 (6.5%)	30 (10.9%)	
	Severe	446 (55.8%)	127 (46.4%)	
	Total	150 (18.8%)	27 (9.9%)	
Vascular access	Femoral	496 (62.1%)	175 (63.9%)	0.613
	Radial	303 (37.9%)	99 (36.1%)	

Angiographic characteristics of the population compared by gender. Abbreviations: IVUS: Intravascular ultrasound, FFR: Fractional flow reserve, OCT: Optical coherence tomography, 95%CI: Mean difference 95% Confidence Interval.

### Use of complementary studies and stent implantation

Complementary studies (FFR, IVUS or OCT) were used in 293 patients (27.3%) and in 3.3% (n = 35) of cases, at least two were necessary. Table 2 describes the complementary studies required in our population. Stents were implanted in 202 (68.9%) patients who underwent complementary studies. Stent implantation was performed in 60.8% (n = 474) of patients in whom complementary studies were not necessary (n = 780). The mean number of stent implants in both groups was 1.25 (SD 1.22) and 1.1 (SD 1.19), respectively *(p* = *0.07)*.

### Factors modifying fluoroscopy time

Fluoroscopy mean (SD) time was 13.8 (11.9) min. We computed two linear multiple-regression models in order to evaluate the factors that affected FT (Table 3). The first model (A) included the number of complementary studies, adjusted by multiple covariates, and the second model (B) evaluated the effect of each complementary study adjusted by multiple covariates:

(A) FT = ß0 + ß1 * Gender + ß2 * DM2 + ß3 * CKD + ß4 * Complementary Studies + ß5 * Stents Placed.
  Where FT = Fluoroscopy time, ß0 = intercept, ß1–5 = Covariates estimates, DM2 = Type 2 diabetes (present/absent, ADA criteria), CKD = Chronic kidney disease (present/absent if Glomerular Filtration Rate < 60 ml/min/1.73m^2^), Complementary Studies = number of complementary studies (1–3), Stent = Number of stents placed.
  Obtaining:
  FT = 6.2 + 1.6 * Gender + 1.9 * DM2–3.2 * CKD + 3.1 * Complementary Studies + *4.2 Stents Placed.

**Table 3:** Linear multiple regression models.

Fluoroscopy time					
	Β	*SE*	*η*	IC 95%	*p-value*
A.*					
Intersection	6.2	0.7	0.058	4.7,7.7	*0.001*
Gender	1.6	0.7	0.004	0.1, 3.0	*0.032*
DM2	1.9	0.6	0.008	0.6, 3.2	*0.004*
CKD	−3.2	1.5	0.004	−6.3, −0.2	*0.038*
Complementary studies	3.1	0.5	0.025	1.9, 4.3	*0.001*
Stents placed	4.2	0.2	0.192	3.7, 4.8	*0.001*
B*					
Intersection	6.2	0.6	0.076	4.93,7.5	*0.001*
Gender	1.5	0.7	0.005	0.18, 2.9	*0.026*
DM2	1.9	0.7	0.007	0.5, 3.3	*0.007*
CKD	−3.2	1.1	0.007	−5.46, −0.9	*0.005*
OCT	2.9	2.2	0.002	−1.4,7.4	*0.183*
FFR	4.6	1.6	0.008	1.5,7.7	*0.004*
IVUS	2.5	0.8	0.009	0.93,4.2	*0.002*
Stents placed	4.2	0.3	0.139	3.65, 4.9	*0.001*
**Radiation dose**					
C*					
Intersection	−1519.4	297.7	0.024	−2103.5, −935.02	0.001
Gender	359.5	98.2	0.012	166.7, 552.3	0.001
BMI	62.6	10.0	0.036	43, 82.2	0.001
HT	205.1	89.1	0.005	30.2, 380	0.022
DM2	181.2	91.2	0.004	3.1, 360.8	0.046
CKD	−581.4	202.2	0.008	−978.1, −184.7	0.004
Lesion severity	138.6	40.3	0.011	59.6, 217.6	0.001
Complementary studies	331.5	192.4	0.003	−46.1, 709.1	0.085
Stents placed	511.9	40.8	0.129	431.8, 591.9	0.001
Lesion severity* Complementary studies**	−187.9	70.8	0.007	−325.7, −49.1	0.008
D*					
Intersection	−1427.2	296.8	0.021	−2009.5, −844.8	0.001
Gender	366.8	98.7	0.013	173.2, 560.4	0.001
BMI	62.3	10.0	0.035	42.6, 81.9	0.001
HT	211.6	89.6	0.005	35.7, 387.4	0.018
DM2	192.2	91.5	0.004	12.7, 371.6	0.036
CKD	−587.6	202.9	0.008	−985.7, −189.4	0.004
Lesion severity	103.9	38.2	0.007	29.0, 178.8	0.007
Number of stents placed	503.1	40.9	0.125	422.8, 583.4	0.001
OCT	−13.03	231.9	0	−468, 442.0	0.954
FFR	−157.3	192.2	0.001	−534.4, 219.7	0.413
IVUS	−154.7	102.2	0.002	−335.2, 45.8	0.130
**Contrast media volume**					
E*					
Intersection	41.9	20	0.004	2.6, 81.14	0.037
Gender	19.1	6.4	0.008	6.5, 31.6	0.003
BMI	1.4	0.7	0.005	0.16, 2.7	0.028
CKD	−52.9	13.1	0.015	−78.6, −27.1	0.0001
Femoral access	30	5.7	0.025	18.8, 41.2	0.0001
Lesion severity	13.9	2.5	0.029	9, 18.8	0.0001
Stents placed	40.6	2.7	0.177	35.3, 45.8	0.0001
Complementary studies	20	5.1	0.014	9.9, 30.1	0.0001
F*					
Intersection	40.1	19.9	0.004	0.8, 79.3	0.045
Gender	18.3	6.4	0.008	5.8, 30.8	0.004
BMI	1.5	0.7	0.005	0.2, 2.7	0.022
CKD	−52.4	13.1	0.015	−78, −26.7	0.0001
Femoral access	31.1	5.7	0.027	19.9, 42.4	0.0001
Lesion severity	14.1	2.5	0.03	9.3, 18.9	0.0001
Stents placed	40.9	2.7	0.18	35.6, 46.1	0.0001
IVUS	8.9	6.7	0.002	−4.2, 22.2	0.182
OCT	39.4	15.2	0.006	9.7, 69.2	0.009
FFR	38	12.5	0.009	13.4, 62.6	0.003

Multiple regression models for factors that predicted Fluoroscopy time (A&B), Radiation dose (C&D) and contrast volume (E&F). Abbreviations: DM2: type 2 diabetes, BMI: body mass index, HT: arterial hypertension, CKD: chronic kidney disease, Complementary studies: Number of Complementary studies, Stents Placed: number of stents placed, lesion severity: Coronary lesion severity B: beta, η: partial eta squared, CI95%: Confidence Interval of 95%,

*Predictive models reached a r^2^ value of A: 0.228, B: 0.228, C: 0.264, D: 0.26, E:0.357 and F: 0.361, respectively. We used a ten-fold cross validation and r^2^ values were A: 0.23, B: 0.22 C:0.36, D:0.25, E: 0.35 and F:0.36 respectively.

**We found interactions between models.
(B) FT = ß0 + ß1 * Gender + ß2 * DM2 + ß3 * CKD + ß4 * OCT + ß5 * FFR + ß6 * IVUS + ß7 * Stents Placed.
  Where FT = Fluoroscopy time, ß0 = intercept, ß1–7 = Covariates estimates, DM2 = Type 2 diabetes (present/absent, ADA criteria), CKD = Chronic kidney disease (present/absent according to Glomerular Filtration Rate < 60 ml/min/1.73m^2^), OCT = (present/absent), OCT = optical coherence tomography (present/absent), FFR = Fractional Flow Reserve (present/absent), IVUS = intravascular ultrasound (present/absent), Stent Placed = Number of stents placed.
  Obtaining:
  FT = 6.2 + 1.5 * Gender + 1.9 * DM2–3.2 * CKD + 2.9 * OCT + 4.6 * FFR + 2.5 * IVUS + 4.2 * Stents Placed

**Figure 1:**
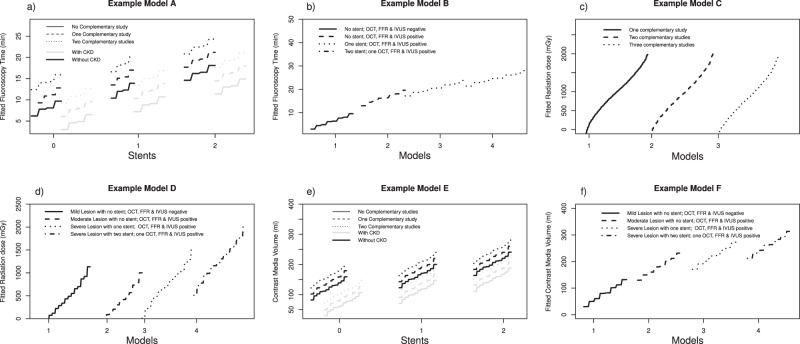
Examples of fitted responses of Fluoroscopy Time, Radiation Dose and Contrast Volume. (**a**) Graphic example of linear multiple-regression model A that evaluates the factors that affected FT. The time increases mainly by the number of stents placed and is reduced in the presence of CKD. The number of complementary studies has a moderate effect. (**b**) Graphic example of linear multiple-regression model B where the main effect of FT was produced by the number of stents. The effect of FFR, OCT and IVUS was minimal. (**c**) Graphic example of linear multiple-regression model C where after adjusting by multiples covariates the number of complementary studies did not affect RD. (**d**) Linear multiple-regression of Model D. The effect of each complementary study with Coronary lesion severity and number of stents adjusted by other covariates was evaluated. The main effect in RD is produced by the number of stents. There is no effect by OCT, FFR and IVUS. (**e**) Example of linear multiple-regression model E, where CV is reduced in patients with CKD and increased when the number of stents rises. The effect of the number of complementary studies is minimal. (**f**) Example of linear multiple-regression model F after adjusting by multiple covariates. The number of stents have high effect con CV; coronary lesion has a moderate effect and each complementary study has a minimal effect.

FFR and IVUS had a small effect on FT (η = 0.008 p < 0.001; η = 0.009, *B* = 2.5, p < 0.001), while OCT had no effect (η = 0.002 *B* = 2.9, p < 0.183). The number of complementary studies performed had a medium effect on FT (η = 0.025, *Β* = 3.1, *p* < *0.001*). Other variables that affected fluoroscopy time were gender; type 2 diabetes and CKD (p < 0.05) Stent implantation had a large effect (η = 0.192, *p* < *0.001*). Fluoroscopy time was similar whether the approach was femoral or right radial (13.6 min vs 13.8 min) *(p* = *0.816)*. Figure 1 letter a and b shows examples of model fitting of FT adjusted by multiple covariates.

### Factor modifying radiation dose

The mean (SD) RD was 1549.58 (1575.6) mGy. We computed two linear multiple-regression models (Table 3) in order to evaluate which factors could affect RD. The first model (C) included the number of complementary studies, adjusted by multiple covariates, and the second model (D) evaluated the effect of each complementary study adjusted by multiple covariates:

(C) RD = ß0 + ß1 * Gender + ß2 * BMI + ß3 * HT + ß4 * DM2 + ß5 * CKD + ß6 * Coronary Lesion Severity + ß7 * Complementary Studies + ß8 * Stents Placed + ß9 * Lesion Severity * Complementary Studies.
  Where RD = Radiation dose, ß0 = intercept, ß1–9 = Covariates estimates, BMI = Boddy Mass Index (kg/m^2^), HT = Hypertension (present/absent, according to systolic blood pressure >140 and/or diastolic > 90) DM2 = Type 2 diabetes (present/absent, according to ADA Criteria), CKD = Chronic kidney disease (present/absent, if Glomerular Filtration Rate < 60 ml/min/1.73m^2^), Lesion Severity = Coronary lesion Severity (1 = vessel occlusion less than 50%, 2 = 50–70%, 3 = 70–995 and 4 = 100%) Complementary Studies = number of complementary studies (1–3).
  Obtaining:
  RD = −1519.4 + 359.5 * Gender + 62.6 * BMI + 205 * HT + 181.9 * DM2–581.4* CKD + 138.6 * Coronary Lesion Severity + 331.5 * Complementary Studies + 511.9 * Stents Placed −187.9 * (Lesion Severity * Complementary Studies).
(D) RD = ß0 + ß1 * Gender + ß2 * BMI + ß3 * HT + ß4 * DM2 + ß5 * CKD + ß6 * Lesion Severity + ß7 * Stents Placed + ß8 * OCT + ß9 * FFR + ß10 * IVUS.
  Where RD = Radiation dose, ß0 = intercept, ß1–10 = Covariates estimates, BMI = Boddy Mass Index (kg/m^2^), HT = Hypertension (present/absent, according to systolic blood pressure >140 and/or diastolic > 90) DM2 = Type 2 diabetes (present/absent, according to ADA Criteria), CKD = Chronic kidney disease (present/absent, if Glomerular Filtration Rate < 60 ml/min/1.73m^2^), Lesion Severity = Coronary lesion Severity (1 = vessel occlusion less than 50%, 2 = 50–70%, 3 = 70–995 and 4 = 100%), Stent Placed = Number of stents placed, OCT = optical coherence tomography (present/absent), FFR = Fractional Flow Reserve (present/absent), IVUS = intravascular ultrasound (present/absent), Stent Placed = Number of stents placed.
  Obtaining:
  RD = −1427.2 + 366.9 * Gender + 62.3 * BMI + 211.6 * HT + 192.2 * DM2–587.6 * CKD + 103.9 * Lesion Severity + 503.1 * Stents Placed – 13 * OCT − 157.3 * FFR − 157.3 * IVUS.

The number of Complementary studies did not affect RD (η = 0.003, *p* = *0.085*). However, it was predicted by the severity of the coronary lesion (η = 0.011, *p* = *0.001)* and the number of stents placed (η = 0.129, *p* = *0.001)*. Gender (η = 0.012, *p* = *0.0001)*, arterial hypertension (η = 0.005, *p* = *0.022)* and type 2 diabetes (η = 0.004*, p* = *0.046)* had a small effect on RD. Chronic kidney disease had a small, negative association with the RD (η = 0.008, *p* = *0.004*). A cubic negative interaction between coronary lesion severity and the number of complementary studies was observed, but the effect was small (η = 0.007, *p* = *0.008*). Figure 1 letter c and d shows examples of model fitting of RD adjusted by multiple covariates.

### Factors modifying contrast volume

The mean (SD) CV used for each procedure was 199.6 (111.1) ml. We computed two linear multiple-regression models (Table 3) in order to evaluate which factors could affect CV. The first model (E) included the number of complementary studies, adjusted by multiple covariates, and the second model (F) evaluated the effect of each complementary study adjusted by multiple covariates:

(E) CV = ß0 + ß1 * Gender + ß2 * BMI + ß3 * CKD + ß4 * Femoral Access + ß5 * Lesion Severity + ß6 * Stents Placed + ß7 * Complementary Studies.
  Where CV = Contrast Volume, ß0 = intercept, ß1–7 = Covariates estimates, BMI = Boddy Mass Index (kg/m^2^), CKD = Chronic kidney disease (present/absent, if Glomerular Filtration Rate < 60 ml/min/1.73m^2^), Femoral Access = Femoral access (yes/no), Lesion Severity = Coronary lesion Severity (1 = vessel occlusion less than 50%, 2 = 50–70%, 3 = 70–995 and 4 = 100%), Stent Placed = %), Stent Placed = Number of stents placed, Complementary Studies = number of complementary studies (1–3).
  Obtaining:
  CV = 41.9 + 19.1 * Gender + 1.4 * BMI − 52.9 * CKD + 30 * Femoral Access + 14 * Lesion Severity + 40.6 * Stents Placed + 20 * Complementary Studies.
(F) CV = ß0 + ß1 * Gender + ß2 * BMI + ß3 * CKD + ß3 * Femoral Access + ß4 * Lesion Severity + ß5 * Stents Placed + ß6 * OCT + ß7 * FFR + ß8 * IVUS.
  Where CV = Contrast Volume, ß0 = intercept, ß1–8 = Covariates estimates, BMI = Boddy Mass Index (kg/m^2^), CKD = Chronic kidney disease (present/absent, if Glomerular Filtration Rate < 60 ml/min/1.73m^2^), Femoral Access = Femoral access (yes/no), Lesion Severity = Coronary lesion Severity (1 = vessel occlusion less than 50%, 2 = 50–70%, 3 = 70–995 and 4 = 100%), Stent Placed = %), Stent Placed = Number of stents placed, OCT = optical coherence tomography (present/absent), FFR = Fractional Flow Reserve (present/absent), IVUS = intravascular ultrasound (present/absent).
  Obtaining:
  CV = 40.1 + 18.3 * Gender + 1.5 * BMI − 52.4 * CKD + 31.1 * Femoral Access +14.1 * Lesion Severity + 40.9 * Stents Placed + 39.4 * OCT + 38 * FFR + 9 * IVUS.

CV was predicted by male gender, BMI, vascular access site, severity of the coronary lesion, the number of placed stents and the performed complementary studies *(p* < *0.05)*. The number of implanted stents had a large effect (η = 0.177, *p* = *0.0001*). Chronic kidney disease had a small effect, decreasing the contrast volume (η = 0.015, *p* < *0.0001*). OCT and FFR had a small effect (η = 0.006, *p* = *0.009 and* η = 0.009, *Β* = 37, *p* = *0.003 respectively)*. Figure 1, letter e and f show examples of model fitting of CV adjusted by multiple covariates.

### Complications and length of hospital stay

Complications occurred in 56 patients (5.2%). Patients with a right femoral approach had more complications compared to those with a right radial approach *(p* < *0.001)*. Complications in the right femoral approach *vs*. the right radial approach were as follows: local hematoma (41 *vs* 5), retroperitoneal hematoma (4 *vs* 0), vascular dissection (2 *vs* 1), thrombosis (1 *vs* 1), and pseudo-aneurysm (1 *vs* 0), respectively. The number of hospitalization days varied according to the vascular approach (4 days *vs* 3.2 days) (p = 0.004).

## Discussion

Our study demonstrated that OCT, FFR and IVUS had a small effect on FT. OCT and FFR had a small effect on CV and none of them had an effect on RD.

The number of FFR, IVUS or OCT in patients who underwent coronary angiography did not affect the RD and had a small effect by increasing FT and CV.

For instance, the best predictors of FT (see Table 3, model A and B) were the number of stent placed and the use of complementary studies; nonetheless these models only predicts 22.8% of the variance of FT; this means that most of the FT (77.2%) is caused by other factors. This contradicts the tendency to attribute complementary studies for excessive FT exposure. The same principle applies to RD (model C and D) and CV (model E and F).

There is increasing evidence supporting the use of complementary studies. In the last decade, the use of IVUS increased six-fold and this tendency continues to rise. In the United States, only 6% of coronary angiographies are guided by IVUS or OCT, while in our population, complementary studies were used in 27.2%^11,12^. The availability of these techniques in our center and actual worldwide trends probably explain our results.

Some authors have reported that complementary studies demand operator expertise, they increase coronary angiography time and, hypothetically, RD and CV^1^. Ionizing radiation is associated with an indirect stress response of the heart, endotheliopathy, atherosclerosis, cancer and *in vitro* apoptosis^5–7^. The risk of nephropathy increases when using contrast media. Because of the frequent use of these techniques and the deleterious events associated with radiation and contrast media, it is important to determine the effect of complementary studies on these variables. Our study found that these complementary studies have a minimal effect on radiation and contrast exposure.

A retrospective study by Ntalianis *et al*. quantified extra angiography time, RD and CV when using FFR in patients who underwent diagnostic angiography^13^. Their results, expressed as a percentage of the entire procedure, showed that FFR used in one artery added an extra 13–26% of FT, 16–30% of RD, and 16–31% of CV, respectively. This study did not include patients undergoing IVUS or OCT, and the interaction between FFR and other radiation and contrast predictors was not assessed (i.e. vascular site access). We included more predictors of radiation and contrast exposure (i.e. lesion severity, number of affected arteries or stents placed) and created multivariate models to observe the effect of IVUS, OCT and FFR on these variables.

Our multivariate models could only predict 22–36% of the variance of FT, RD and CV; this means that other predictors must be measured in the future (Table 3).

This study demonstrated that complementary studies explain only a small percentage of the radiation and contrast media variations when used in coronary angiography. There are many advantages to complementary studies such as decreasing mortality, guidance in stent placement, and better angiography outcomes^3,14^. Patients can avoid unnecessary stent placement.

The number of stents placed was the only variable that showed a strong association with RD, CV and FT, so complementary studies are safe in daily practice.

Male sex was associated with greater radiation and contrast medium exposure even when men were younger than women and had less type 2 diabetes mellitus and arterial hypertension. Arterial hypertension, type 2 diabetes mellitus, BMI and severity of the coronary lesion were associated with a greater CV and RD. This may be due to the fact that these comorbidities are associated with diffuse atherosclerosis, complex plaque characteristics (i.e. diffuse lesions), increased angiography complexity and complication development^15–17^. Although CKD was present in a small sample (n = 49), this was the only factor that showed a negative association with FT, RD and CV, but more studies are needed to reach a definite conclusion.

A radial vascular approach has been previously associated with greater RD and FT than the femoral approach^9^. In our models, a femoral approach did not influence the radiation dose or fluoroscopy time but exposed patients to greater CV. The use of the radial artery approach is increasingly more common because of the low complication rate (i.e. hematoma and bleeding) which represents an additional advantage^18^. The femoral vascular approach was associated with a greater number of complications and a prolonged in-hospital stay, as reported in previous studies^19^.

### Study limitations

Although this study represents a real-life study, the lack of randomization can lead to selection bias. Another important limitation is that inter-operator variability in coronary angiography (an operator-dependent procedure) was not measured, and one can assume that operator expertise is a factor associated with radiation and contrast exposure.

## Conclusion

Complementary studies did not increase RD and had a small effect on FT and CV. Stent placement is the factor that most increased radiation and contrast medium exposure.

## Data Availability

Data base are available upon request
